# Anterior Open Bite Malocclusion: From Clinical Treatment Strategies towards the Dissection of the Genetic Bases of the Disease Using Human and Collaborative Cross Mice Cohorts

**DOI:** 10.3390/jpm13111617

**Published:** 2023-11-17

**Authors:** Iqbal M. Lone, Osayd Zohud, Kareem Midlej, Eva Paddenberg, Sebastian Krohn, Christian Kirschneck, Peter Proff, Nezar Watted, Fuad A. Iraqi

**Affiliations:** 1 Department of Clinical Microbiology and Immunology, Sackler Faculty of Medicine, Tel-Aviv University, Tel-Aviv 69978, Israel; iqbalzoo84@gmail.com (I.M.L.); osaydzohud@mail.tau.ac.il (O.Z.); kareemmidlej@mail.tau.ac.il (K.M.); 2 Department of Orthodontics, University Hospital of Regensburg, D-93053 Regensburg, Germany; eva.paddenberg@ukr.de (E.P.); sebastian.krohn@klinik.uni-regensburg.de (S.K.); peter.proff@klinik.uni-regensburg.de (P.P.); 3 Department of Orthodontics, University of Bonn, D-53111 Bonn, Germany; christian.kirschneck@uni-bonn.de; 4 Center for Dentistry Research and Aesthetics, Jatt 45911, Israel; nezar.watted@gmx.net; 5 Department of Orthodontics, Faculty of Dentistry, Arab America University, Jenin 919000, Palestine; 6 Gathering for Prosperity Initiative, Jatt 45911, Israel

**Keywords:** malocclusion, open bite, etiology, treatment, Collaborative Cross mice

## Abstract

Anterior open bite malocclusion is a complex dental condition characterized by a lack of contact or overlap between the upper and lower front teeth. It can lead to difficulties with speech, chewing, and biting. Its etiology is multifactorial, involving a combination of genetic, environmental, and developmental factors. Genetic studies have identified specific genes and signaling pathways involved in jaw growth, tooth eruption, and dental occlusion that may contribute to open bite development. Understanding the genetic and epigenetic factors contributing to skeletal open bite is crucial for developing effective prevention and treatment strategies. A thorough manual search was undertaken along with searches on PubMed, Scopus, Science Direct, and Web of Science for relevant studies published before June 2022. RCTs (clinical trials) and subsequent observational studies comprised the included studies. Orthodontic treatment is the primary approach for managing open bites, often involving braces, clear aligners, or other orthodontic appliances. In addition to orthodontic interventions, adjuvant therapies such as speech therapy and/or physiotherapy may be necessary. In some cases, surgical interventions may be necessary to correct underlying skeletal issues. Advancements in technology, such as 3D printing and computer-assisted design and manufacturing, have improved treatment precision and efficiency. Genetic research using animal models, such as the Collaborative Cross mouse population, offers insights into the genetic components of open bite and potential therapeutic targets. Identifying the underlying genetic factors and understanding their mechanisms can lead to the development of more precise treatments and preventive strategies for open bite. Here, we propose to perform human research using mouse models to generate debatable results. We anticipate that a genome-wide association study (GWAS) search for significant genes and their modifiers, an epigenetics-wide association study (EWAS), RNA-seq analysis, the integration of GWAS and expression-quantitative trait loci (eQTL), and micro-, small-, and long noncoding RNA analysis in tissues associated with open bite in humans and mice will uncover novel genes and genetic factors influencing this phenotype.

## 1. Introduction

Malocclusion, a common dental condition impacting many individuals worldwide, refers to the misalignment of the teeth and jaws. This condition can adversely affect oral health, impairing eating, speaking, and the maintenance of good oral hygiene. Moreover, malocclusion can influence appearance, diminishing self-confidence and self-esteem [1]. The causes of malocclusion are multifactorial and intricate, involving genetic, environmental, and developmental factors [1]. Dental research has made significant progress in understanding the molecular mechanisms underlying malocclusion, particularly through exploring genetics and genomics. These studies have revealed specific genes and signaling pathways that influence jaw formation, tooth emergence, and tooth closure. However, fully comprehending the intricate interplay between hereditary and environmental variables which leads to malocclusion remains a challenging task [2].

**Figure 1:**
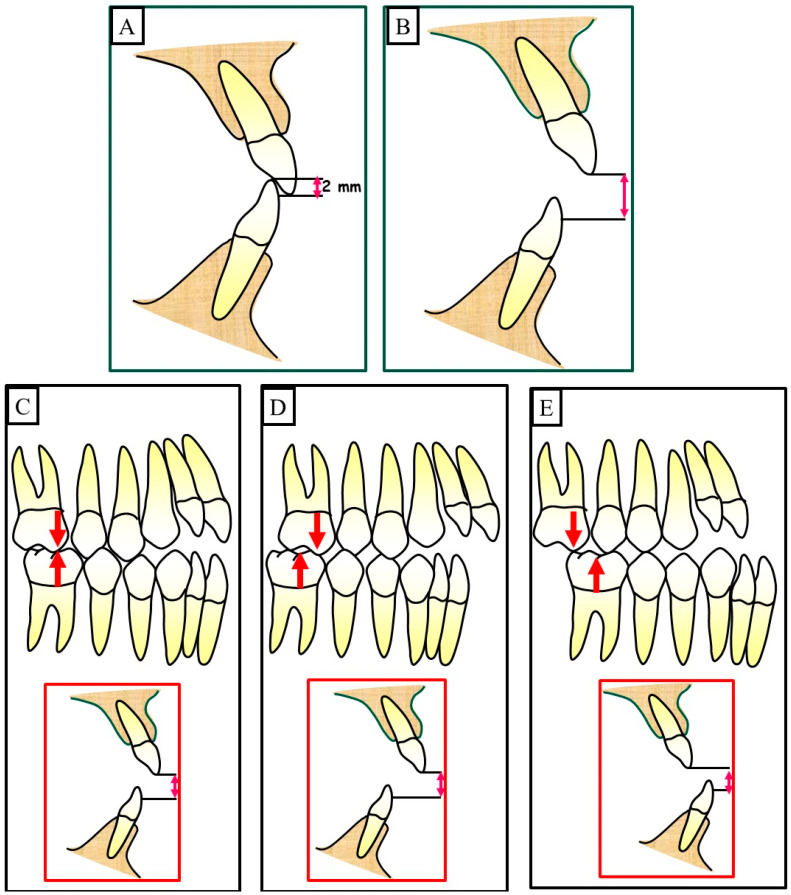
Overbite denotes the vertical alignment or the space between the upper central incisor of the maxilla and the corresponding central incisor of the mandible. (**A**) A normal or physiological overbite typically measures about 2–3 mm. (**B**) An open bite refers to a reduced overbite, usually measuring less than 0 mm. (**C**) Open bite combined with a Class I dental relationship. (**D**) Open bite in association with a Class II dental relationship. (**E**) Open bite in connection with a Class III dental relationship.

**Figure 2:**
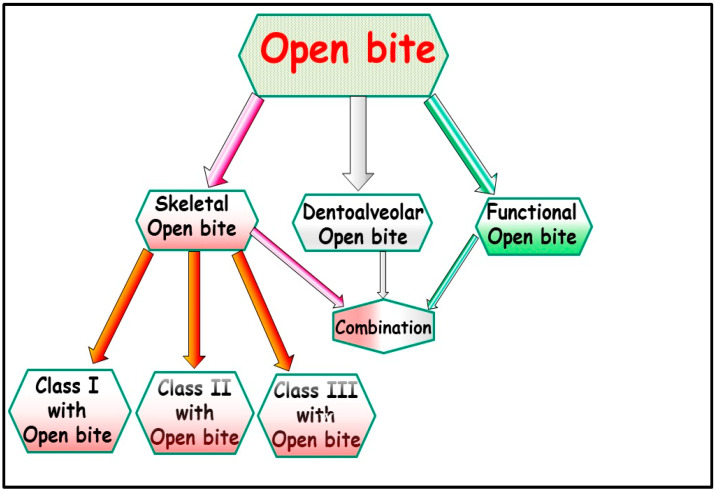
Various categories of open bites exist, and a visual representation in the form of a diagram can help elucidate these distinctions.

**Figure 3:**
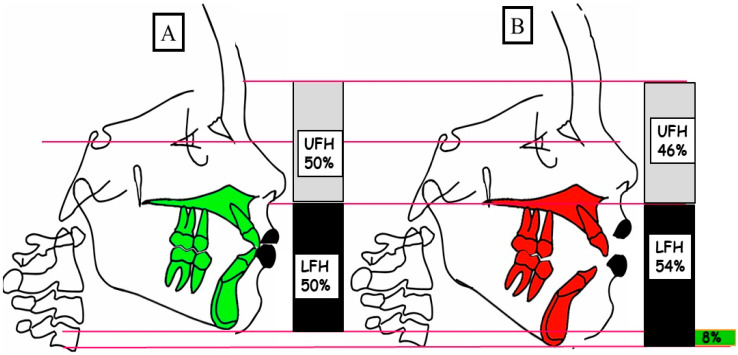
A schematic depiction of the vertical dimension illustrates a physiological overbite (**A**) and a skeletal open bite (**B**). (**A**) In a physiological vertical dimension, there is a balanced relationship between the upper facial height (UFH 50%) and lower facial height (LFH 50%). (**B**) A skeletal open bite is characterized by an elevated lower facial height (LFH 54%) in comparison to the upper facial height (UFH 46%).

**Figure 4:**
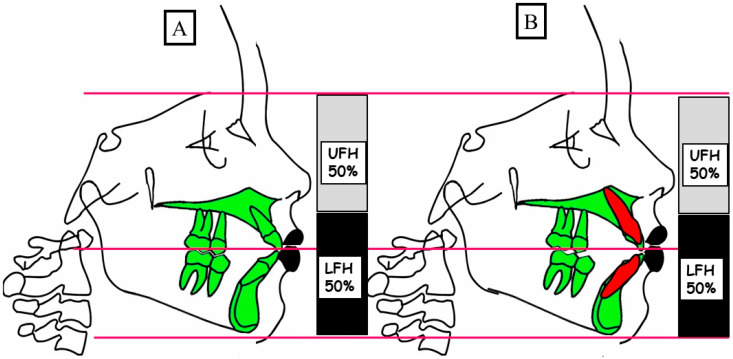
A schematic illustration of the vertical dimension showcases a physiological overbite (**A**) and a dentoalveolar open bite (**B**). (**A**) In a physiological vertical dimension, there exists a harmonious relationship between the upper facial height (UFH 50%) and lower facial height (LFH 50%). (**B**) A dentoalveolar open bite occurs due to the infraocclusion or protrusion of the front teeth, maintaining a balanced relation between the upper facial height (UFH 50%) and lower facial height (LFH 50%).

**Figure 5:**
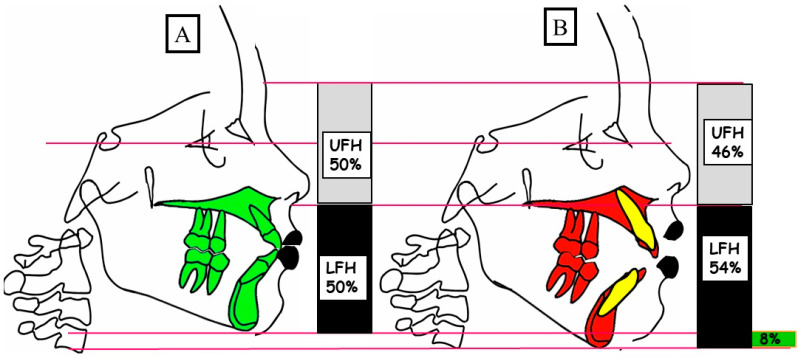
A schematic portrayal of the vertical dimension demonstrates a physiological overbite (**A**) and the coexistence of a skeletal and dentoalveolar open bite (**B**). (**A**) In a physiological vertical dimension, there is an equilibrium between the upper facial height (UFH 50%) and lower facial height (LFH 50%). (**B**) A combined skeletal and dentoalveolar open bite results from the posterior rotation of the mandible and the infraocclusion of the front teeth, leading to an elevated lower facial height (LFH 54%) in contrast to the upper facial height (UFH 46%).

**Figure 6:**
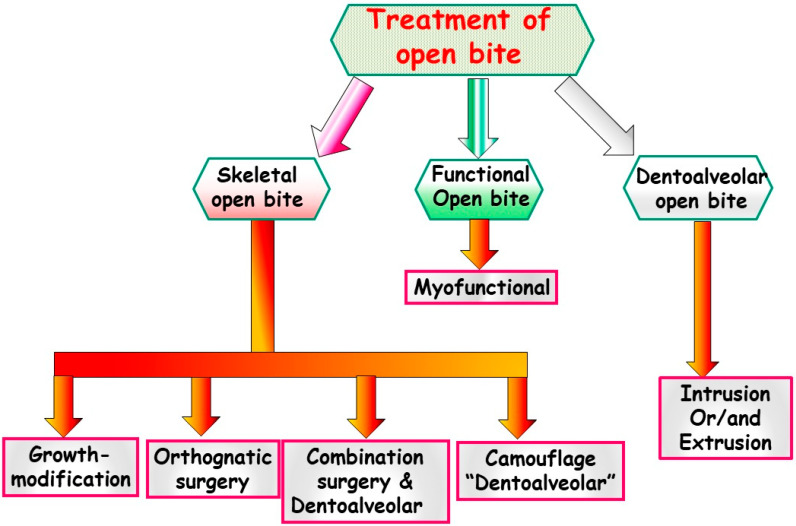
A diagram depicting various treatment approaches for open bite, which can be categorized based on factors such as age, growth stage, causative factors, functional considerations, and aesthetic concerns.

Anterior open bite is a type of malocclusion that occurs when there is no contact or overlap between the upper and lower front teeth. It can also occur with all forms of skeletal Dysgnathy (Figure 1A–E). Prolonged use of a pacifier may contribute to its development. It is a complex dental problem that can be triggered by a number of circumstances, such as genetics, environmental factors, and habits such as thumb-sucking or tongue-thrusting [3]. An open bite can be classified as either functional, skeletal, or dentoalveolar, or as a combination of all three, depending on the underlying cause (Figure 2). Skeletal open bite is caused by excessive vertical growth of the dentoalveolar complex, especially in the posterior molar region. In this patient category, there is an increased lower face height compared to the upper face height (long face syndrome). Cephalometric open bite is a hyperdevirgence of the InterBase angles (angles between the upper and lower jaw base). This long face can be seen extra-orally (Figure 3A,B). At the same time, dental open bite is generally found in the anterior region within the area of the cuspids and incisors and is associated with a normal craniofacial pattern, proclined and undererupted anterior teeth, and thumb- or finger-sucking habits. In this patient category, there is a harmony between the upper and lower facial height. Nothing is noticeable extra-orally, as shown in Figure 4A,B [3]. The third category consists of the combination of skeletal and dentoalveolar open bite, in which the intraoral and the extra-oral symptoms can be seen (Figure 5A,B). An open bite can cause difficulties with speech, chewing, and biting and can also lead to temporomandibular joint (TMJ) disorders [4]. Treatment of an open bite can be challenging and may require a combination of orthodontic and surgical interventions (Figure 6). This article reviews the etiologies, dentofacial morphology, treatment modalities, retention, and stability of anterior open bite [3].

## 2. Top of Form

Open bite malocclusion can be classified into different types based on its location and etiology. According to Moyer, simple open bite is limited to the teeth and their surrounding alveolar process, whereas a complex open bite is primarily the result of vertical dysplasia and is often linked to Class I, Class II, and Class III malocclusions (Figure 1A–E and Figure 2). False or dental open bite is characterized by proclined teeth without alteration of the osseous bases, while true or skeletal open bite involves deformed alveolar processes and dolichofacial characteristics. Posterior open bite is defined as the failure of contact between the posterior teeth when the teeth occlude in centric occlusion. Open bites can also be classified based on their location, such as anterior open bite, which can be dental or skeletal, and posterior open bite. The classification of open bite includes simple open bite, compound open bite, and infantile open bite. Understanding the different types and classifications of open bite is crucial in diagnosing and planning treatment for such cases [5].

Strong pieces of evidence suggest that genetics and heredity can play a significant role in the development of open bite malocclusion. Evolution has led to genetically determined smaller jaws and more vertical facial structures, which can increase the risk of open bite. Height may also be a hereditary factor, and was shown to be a coounder involved in affecting understanding of the genetic and environmental variables that contribute to open bite, which are critical for precise diagnosis and therapy [6].

The utilization of animal models has greatly contributed to the advancement of molecular analysis in the field of malocclusion research. By leveraging these models, researchers are able to delve into the intricate interplay between environmental and genetic variables that possess a role in the formation of tooth misalignment, as well as to explore and evaluate diverse preventive and therapeutic strategies. Among the various animal models available, mice have emerged as a particularly advantageous choice due to their small physical stature, ease of breeding, and remarkable genetic resemblance to humans [7].

An animal model that has proven to be highly effective in investigating complex genetic traits like malocclusion is the Collaborative Cross (CC) mouse. Through meticulous breeding techniques, CC mice can give rise to a genetically diverse population, which considerably expands the range of genetic variations available for comprehensive examination. This particular mouse population serves as an exceptional model for the thorough investigation of the intricate genetic underpinnings associated with malocclusion, owing to its possession of a distinct and heterogeneous assortment of genetic variants [7]. Notably, CC mice have been successfully harnessed in previous studies to unravel the complexities underlying a wide array of genetic traits, including but not limited to body mass index and metabolic diseases [8]. Consequently, the inherent genetic diversity present within the CC mouse population offers an invaluable resource for gaining a comprehensive understanding of the multifaceted genetic components implicated in malocclusion, thereby facilitating the targeted identification of specific genetic variants that contribute to the manifestation of complex traits [9]. The role of genetic factors in the emergence of open bite will be addressed in this paper, and the potential of the CC mouse population in identifying genetic variants that contribute to open bite will be highlighted. We also suggest that the implications of this proposed research will lead to the development of precise treatments and preventive strategies for open bite. The use of the CC mice cohort is pivotal in our research. This unique mouse model, created through meticulous breeding, replicates the genetic diversity encountered in human populations. CC mice have proven effective in unraveling the complexities of diverse genetic traits, offering a dynamic platform for genetic studies. By harnessing the CC mice, we aim to simulate and understand the genetic components of open bite and identify specific genetic variants which contribute to its manifestation.

## 3. Etiology

The etiology of open bite is complex and multifactorial, involving a combination of genetic, environmental, and developmental factors. Interaction between genetic and environmental factors can influence open bite development. For example, a genetic predisposition to malocclusion combined with oral habits such as thumb-sucking can lead to more severe malocclusion. Understanding these factors and their interactions is critical for developing effective prevention and treatment strategies for open bite [10]. Genetic factors are believed to play a significant role in the development of open bite. Studies have identified specific genes and signaling pathways implicated in the growth of the jaw and the eruption of teeth, and variations in these genes may contribute to open bite development.

Genetic factors do not solely influence the development of malocclusion, as epigenetic mechanisms also contribute to its manifestation. Epigenetic processes, encompassing DNA methylation, histone modification, and microRNA regulation, have been identified as influential elements in the intricate web of malocclusion development. These epigenetic changes possess the ability to modify patterns of gene expression, thereby impacting the growth and development of teeth and jaws. Research has demonstrated that patients with malocclusion exhibit altered DNA methylation patterns, which, in turn, can disrupt transcriptome profiles that are crucial for the development of the jaw and the emergence of the teeth [11]. Furthermore, it is important to note that environmental factors, including diet, stress, and exposure to toxins, can influence epigenetic modifications. For instance, research has revealed that maternal stress experienced by mothers throughout gestation can cause changes in DNA methylation patterns in children, eventually leading to craniofacial deformities and malocclusion [12].

To summarize, the bases for malocclusion development extend beyond genetic differences alone. They also involve intricate interactions between genes, signaling pathways, and epigenetic factors. The genetic factors encompass variations in genes responsible for mandibular development, the eruption of teeth, and oral occlusion. However, epigenetic mechanisms, including DNA methylation and microRNA control of microRNA activity, exert their influence on malocclusion development as well. By comprehensively gaining insights into the genetic and epigenetic elements that play a role in the development of malocclusion, researchers can provide the foundation for the creation of innovative preventative and therapeutic solutions aimed at addressing this prevalent dental condition.

## 4. Classification of Open Bite

### 4.1. Anterior Open Bite

**Figure 7:**
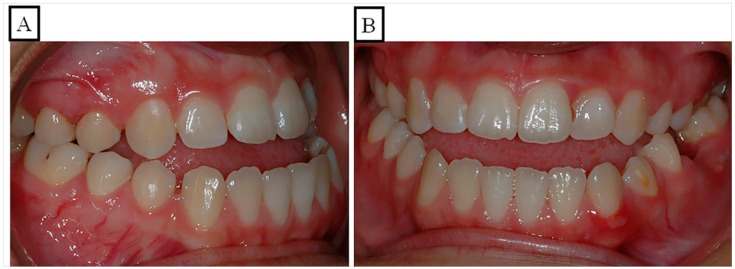
An anterior portion of the dental arches without contact is meant by a malocclusion in this context (**A**); (**B**) the posterior teeth in occlusion.

A malocclusion, in this context, refers to an anterior portion of the dental arches without any contact, and the posterior teeth in occlusion are shown in Figure 1A–E and Figure 7A,B. When a malocclusion affects the posterior segment, it is referred to as a combined open bite [13]. Anterior open bite is one of the most widespread and challenging to treat of the malocclusions that are most frequently encountered in clinic practice. When the etiology is multifactorial, the pathology results in aesthetic changes, harm to the articulation of some phonemes, and unfavorable psychological states [14,15]. Functional, dentoalveolar, skeletal, or a combination of other factors may be the cause of the open bite. Using fixed appliances for orthodontic treatments makes treating dental open bite simple. For the treatment of skeletal open bite, which may necessitate orthognathic surgery, a more thorough strategy is needed. Myofunctional appliances can be used to repair dental open bite in growing patients, and afterward, orthodontic removable appliances can be used to fix it during the retention phase [16]. The prepubertal and pubertal growth should be evaluated for nasal obstruction [17]. The axial inclinations of the incisors might change as a result of excessive activity of the tongue while swallowing or even when it is at rest, which may cause an open bite [18].

### 4.2. Posterior Open Bite

**Figure 8:**
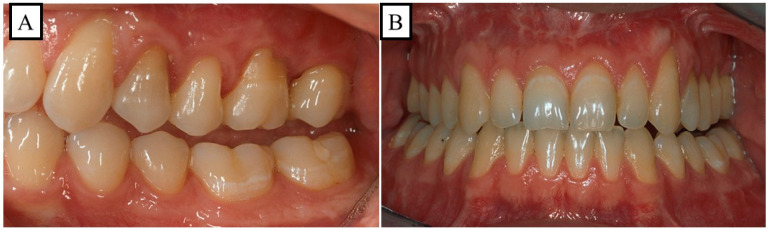
Posterior open bite is the loss of contact between the posterior teeth. Subfigure (**A**) shows the posterior open bite from first premolar to the second molar, and (**B**) the same patient shows contact in anterior area, while open in the posterior area.

When the teeth occlude in centric occlusion, posterior open bite is the loss of contact between the posterior teeth, as shown in Figure 8A,B. The maxillary and mandibular premolars are not occluded, as seen in the figures. Insignificant contact exists between the mandibular and maxillary molars in a typical anterior overjet and overbite.

### 4.3. Types of Open Bite

#### 4.3.1. False or Dental Open Bite

**Figure 9:**
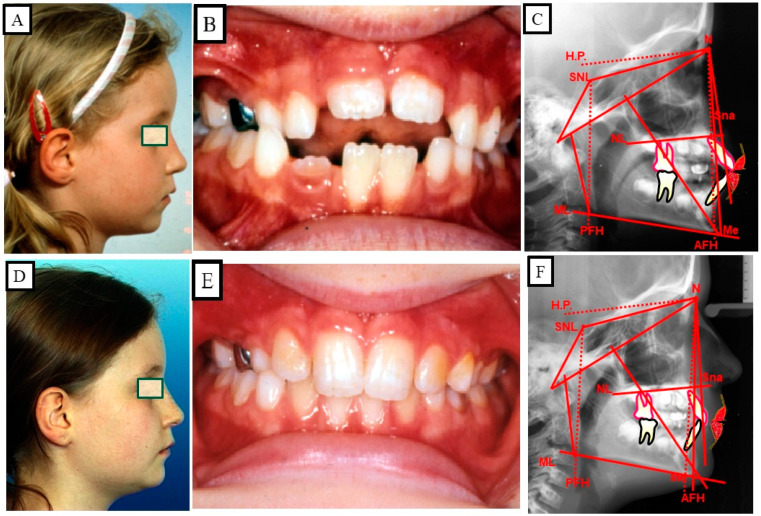
False (**A**) or dental (**B**) open bite. (**A**–**C**) During tooth development and tooth eruption. (**D**–**F**) After tooth development and complete tooth eruption.

The osseous roots of the teeth in this bite are unaltered, and the procline does not reach past the canine. This individual displays a pseudo-bite, a dentoalveolar issue, normal facial morphology, and proper bone relationships, all of which are shown in Figure 9A–F and fully discussed by Meyer-Marcotty et al. and Rodriguez and Casasa [19,20].

#### 4.3.2. True or Skeletal Open Bite

Alveolar processes that are implicated or malformed as well as dolichofacial characteristics are found in this kind of open bite. The lower third and vertical dimensions of this patient’s face are increased, and they have hyper-divergency in their maxilla, as shown in Figure 3A,B and Figure 5A,B and discussed by Chang and Moon [21].

Open bites are classified into anterior and posterior open bites based on the region in which they occur. From an etiological standpoint, these two divisions correspond to dental and skeletal open bites. A dental eruption obstruction causes the dental anterior open bite, while posterior facial development causes the skeletal open bite. The posterior open bite is defined by the inability of a significant number of teeth in either or both of the opposing buccal segments to achieve occlusion despite incisor contact. It occurs seldom and may be brought on by the tongue interposing, eruptional disturbances (such as ankylosis), a basic lack of eruption, or a fully open bite, as presented in Figure 5A,B and Figure 7A,B and discussed by Greenlee et al. [22].

### 4.4. Andrew Richardson Classification

#### 4.4.1. Transitional Open Bite

When the permanent teeth start erupting, this kind of open bite happens. An anterior open bite is the outcome of the dentoalveolar region’s inadequate development. Alveolar growth that continues and the typical growth increase in the lower anterior face height cause spontaneous adjustments.

#### 4.4.2. Digit-Sucking Open Bite

**Figure 10:**
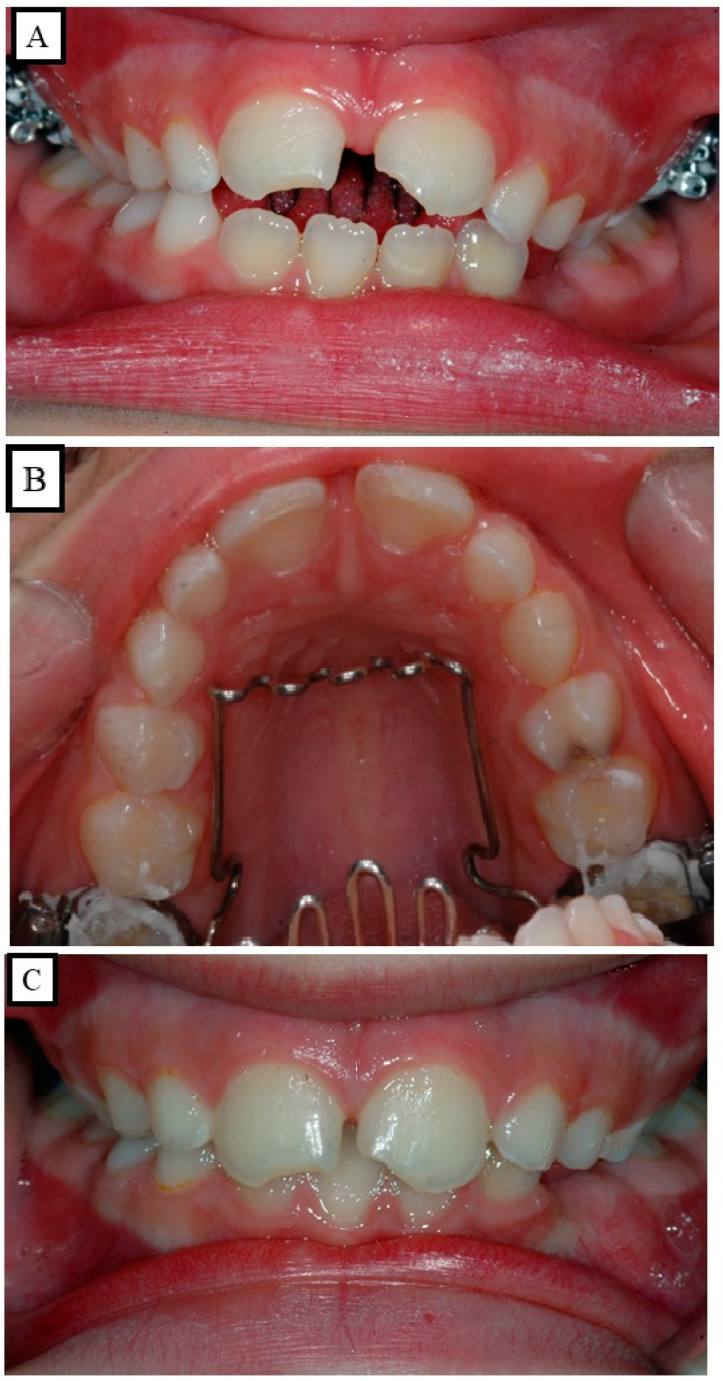
An anterior open bite results from digit sucking. After preventing finger sucking by using a special device, spontaneous closure of the anterior open bite occurred (**A**–**C**).

An anterior open bite results from digit sucking, which prevents the incisor teeth from erupting fully. By stopping the habit, such open bites can be avoided (Figure 10A–C). Rarely do these open bites last throughout adulthood, despite being uncommon during pubertal growth stages. Anterior open bites often spontaneously close due to the growth of the dentoalveolar process and the incisor uprighting. Cysts, dilacerations, and ankylosis are a few of the local pathological diseases that can cause an anterior open bite. A proper surgical procedure to remove the local disease facilitates dentoalveolar development. In contrast, an open bite brought on by the skeletal pathology or abnormalities becomes visible near the conclusion of the growing phase. These illnesses include cleft palate, craniofacial dysostosis, cleidocranial dysostosis, and achondroplasia. The nonpathological skeletal group is divided into three subgroups.

Open bite during the early tooth development phase which closes throughout growth periods before and during puberty is caused by dentoalveolar growth compensation. As a result, the frequency of open bites tends to decline with age. The second subgroup is noticeable in the pre-pubertal period, but it disappears throughout adolescence and reemerges during the post-pubertal stage. This is a result of the interaction between the vertical facial growth and compensatory dentoalveolar growth, which is enough to seal the open bite. However, vertical facial growth takes over in the post-pubertal period and results in an open bite. The third category poses the most challenging clinical orthodontic issue, as facial development predominates and causes a gross anterior open bite as people mature [23].

### 4.5. Factors and Characteristics of Open Bite

One of the most challenging orthodontic issues is open bite malocclusion, which is regarded as a problem. Multiple variables, including genetic and environmental influences, can contribute to open bites. Two general categories—skeletal and dental—can be used to classify open bites. A real skeletal open bite might require dental surgery in addition to orthodontic therapy, while a dental open bite can be corrected with braces. An open bite can cause people to experience problems with their appearance, oral function, and mental health. Children’s development is hampered by functional issues, which include speech, mastication, and deglutition defects. The open bite frequency might reach up to 17% in mixed dentition [24]. Recurrent adenoid infections can result in a malpositioned tongue, a chronic infantile swallow, and bad oral habits that can be observed as the incisors are partially erupting. Dento-alveolar anterior open bite is caused by bad oral practices such as finger or lip sucking, breathing through the mouth, and tongue pushing. This is easily remedied with just orthodontic care. This is true if the patient receives a diagnosis early and the related practices can be changed. Mouth breathing is frequently accompanied by difficulty in speaking, particularly spitting consonants, and dry nasal passages. There is a propensity for vertical dentofacial dysplasia to recur. Both open bite and deep bite malocclusions result in this [25]. Anterior open bite found in vertical dysplasia is multifactorial [26,27].

### 4.6. Hereditary Factors

Most frequently, inherited facial expansion is connected to the open bite abnormality. Dysplasias in the vertical plane may be inherited since horizontal skeletal dysplasias appear to be inherited [28]. Research on the causes of craniofacial growth have focused on three significant ideas in the last few years [29]. Similar to other tissues, bone determines its own growth to a large extent [30]. Cartilage controls skeletal development, with the bone acting incidentally and passively [31].

### 4.7. Non-Hereditary Factors

Maciel and Leite [32] and Arat et al. [33] have emphasized aberrant functioning patterns and harmful oral habits of the tongue, as well as atypical swallowing habits (Figure 10A–C) and speech issues, as contributing to and being part of the open bite phenomena. An irregular swallowing pattern may be the root cause or the outcome of a tongue problem.

The location of the open bite defect varies depending on the pressures present and the teeth and supporting structures’ capacity to resist change, according to Wajid et al. [5]. There may be a propensity toward an anterior open bite, for instance, if the swallowing pattern is incorrect and the tongue is propelled forward strongly. Additionally, the existence of harmful thumb, finger, or lip suckling, as well as poor oral breathing practices and weak labial muscles, have a significant impact on the severity of the anterior open bite.

## 5. Sucking Habits

**Figure 11:**
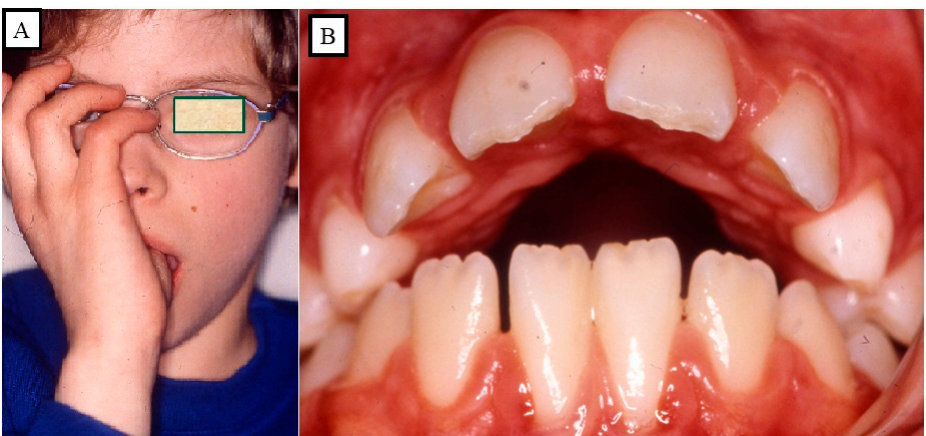
An anterior open bite (**A**), the upper jaw shape results from thumb sucking during growth (**B**).

The duration, frequency, severity, and location of the sucking practices all play a role in the extent of the harm done to the teeth and underlying tissues. When a child is between the ages of four and five, thumb- or finger-sucking habits might be observed. This is regarded as a typical habit that does not cause a malocclusion to become permanent. An anterior open bite, however, may well occur if thumb sucking continues unabated up until the age groups of mixed and permanent dentition [34]. Some youngsters actively suck their thumbs or fingers, while others just let their thumbs passively lie in their mouths. The malocclusions will have varied degrees of severity depending on the intensity and consistency of the habit. The anterior aspect of the maxillary complex may be subjected to upward and forward stress as a result of frequent thumb sucking (Figure 11A,B).

## 6. Adaptability

According to Reichert et al. [35], an open bite with ineffective lips may occur from an excessive rearward rotation of the mandible. For the purpose of forming an oral seal during deglutition, hyperactive mentalis and tongue muscles may be needed [36].

## 7. Environmental Factors

**Figure 12:**
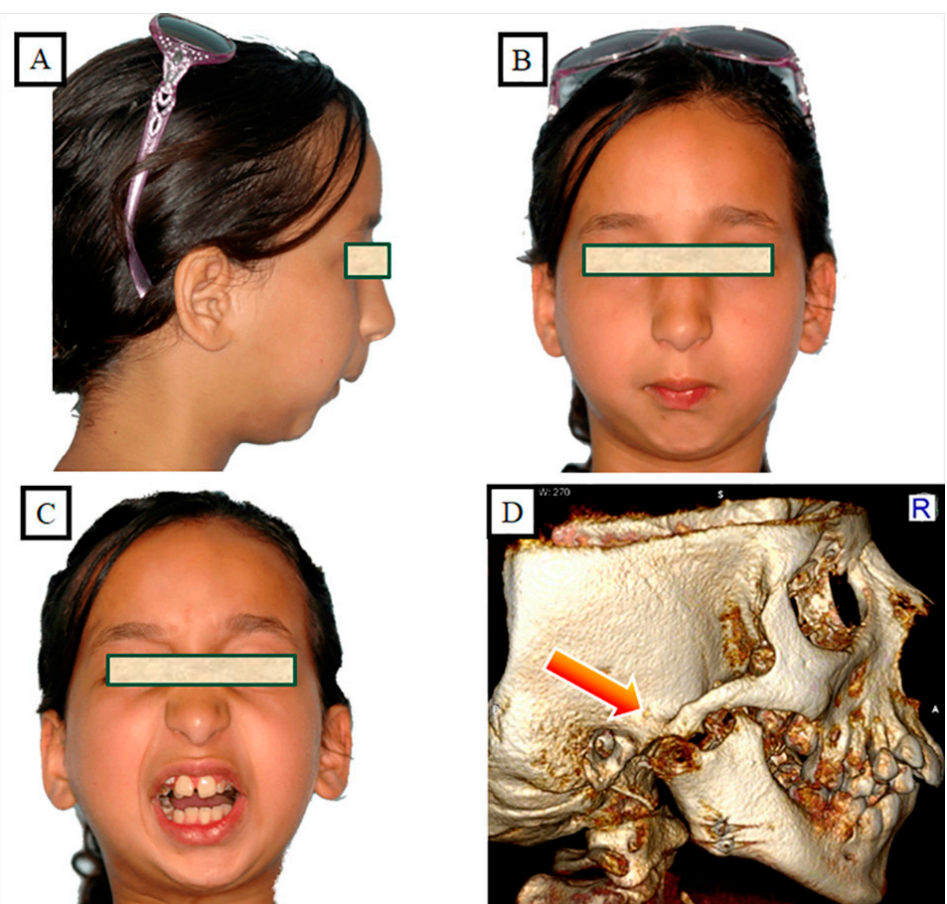
Patient with a temporomandibular joint fracture in the growth phase, the result was ankylosis and growth disorders in the ramus mandible, an open bite developed (**A**–**D**).

Habits, neurological impairments, trauma, and illnesses are examples of environmental variables. According to Park et al. [37], macroglossia is frequently accompanied by harmful oral behaviors such as tongue thrusting and mouth breathing. According to Togawa et al. [38], the neuromuscular inadequacies classify the open bite’s skeletal component. Leptoprosopic patients with muscular dystrophy exhibit supra eruption of the posterior buccal segment, precipitating as an anterior open bite. Skeleto-facial or dentoalveolar trauma are two possible types. Ankylosis of the condyle, which manifests as abnormal vertical growth of the mandible, or the stoppage of condyle growth, which causes a clearly defined anterior open bite, are the most common causes of this condition (Figure 12A–D). An anterior open bite is a sign of dental trauma, especially to the incisors. Prior to the patient’s full growth, damaged teeth begin to ankylose [39]. Condylar resorption frequently coexists with degenerative conditions such as idiopathic condylar resorption and juvenile rheumatoid arthritis [40].

## 8. Genetics

The genetic make-up of the body controls innate growth potentials. For instance, control over the sagittal, transverse, and vertical dimensions is typically passed on in families, like the Hapsburg jaw. The patient’s genetic makeup is also responsible for growth and growth rotations that take place in the late maturation stage. Molars can erupt vertically in facial types like the hyper and leptoprosopic types, which results in an excessively vertical skeletal architecture [41].

## 9. Findings of Literature Search

Although the etiology of posterior open bite (POB) is poorly understood, a possible genetic cause has been suggested in an investigation of a non-syndromic family collection of cases with strong POB penetrance over two generations [42]. The intricate interaction of environmental and genetic variables contributes to the root cause of POB, a complex issue to understand and comprehend. However, the possibility of a genetic cause for POB exists [42]. The majority of genetic investigations on the genesis of malocclusion have been on syndromic disorders. Many single-gene correlated diseases, such as Apert’s syndrome, Treacher Collins syndrome, and cleidocranial dysplasia, are characterized by craniofacial and oral symptoms and are triggered by alterations in fibroblast growth factor receptor 2 (FGFR2), treacle ribosome biogenesis factor 1 (TCOF1), and Runt-related transcription factor 2 (RUNX2), correspondingly [42]. Malocclusions in these illnesses tend to be components of and subsequent to a complicated pattern of several dentofacial abnormalities. Although there has been no evidence in the literature of any genetic etiology of POB (excluding those indirectly induced by primary failure of eruption (PFE)), the potential remains [42]. There is an unusual pedigree of POB patients with strong penetrance spanning two generations in the orthodontic clinic at Rutgers School of Dental Medicine, but no syndromic disorders have been recorded. Patients MM1, MM2, and MM3 are three Caucasian siblings who all have POB. They all have straight to somewhat concave profiles, perioral muscular tension, and several missing teeth. The study implies that identifying the implicated gene(s) and their roles will assist scientists in comprehending the etiology and processes of POB, as well as in building the groundwork for improved therapies and results [42]. This article highlights the importance of identifying the fundamental genetic component of POB by genetic linkage analysis or whole genome sequencing to better understand its mechanisms. Further investigations into the gene(s) and mechanism(s) implicated cannot just offer an exceptional chance to better comprehend POB and the complex muscular–occlusal interaction, it can also provide strong insight into the most successful treatments.

Several studies have investigated the genetic factors contributing to this malocclusion [43]. One of the main findings of these studies is that open bite is a polygenic characteristic, indicating that it is caused by the simultaneous segregation of many genes [43]. The specific genes leading to a particular skeletal variability are not yet fully understood, but studies have demonstrated that vertical characteristics are more genetically regulated than anteroposterior parameters, and heredity is displayed anteriorly rather than posteriorly [43]. The mandibular shape also seems to be determined more genetically than mandibular size. Different inheritance models have been suggested for mandibular prognathism, which is sometimes associated with open bite, including the simple recessive or autosomal dominant with incomplete penetrance models. However, the polygenic nature of craniofacial features makes determining the precise genes responsible for skeletal variations difficult [43]. In addition to genetic factors, environmental factors such as thumb sucking, tongue thrusting, and mouth breathing can also lead to the formation of open bite. However, the relative contribution of genetic and environmental factors to open bite development is not yet fully understood [43]. A genetic syndrome might accompany some malocclusions with severe skeletal discrepancies, and mutations in specific genes can cause these syndromes. For example, mutations in the Fibrillin (FBN) 1 gene are the major cause of Marfan syndrome, which can lead to maxillary/mandibular retrognathia, a long face, a highly arched palate, and other craniofacial abnormalities. In conclusion, the genetic basis of open bite is complex and not fully understood. Open bite is a polygenic trait, and the specific genes leading to particular skeletal variabilities are not yet fully understood. Further genetic investigations are needed to identify all of the individual genes that contribute to certain skeletal variations, which could pave the way to the genetic repair of genetically regulated dentofacial malformations and malocclusions in the future [43].

Another research article explores the relationship between amelogenesis imperfecta, which is a genetic disorder that affects the development of tooth enamel, which is the hard, outer layer of the teeth. This condition can cause teeth to be discolored, to be pitted, or to have an abnormal shape. In some cases, the enamel may be so thin that the teeth are more prone to damage or decay. Amelogenesis imperfecta can be inherited in an autosomal dominant, autosomal recessive, or X-linked pattern, and there are several different types of the condition with varying degrees of severity, including anterior open bite, a dental condition where the front teeth do not touch. The study found that an anterior open bite was always associated with a severe discrepancy in the vertical relationship of the jaws and that this vertical dysgnathia was the primary etiological factor predisposing patients to an anterior open bite. The article suggests that the frequent association of anterior open bite and amelogenesis imperfecta is caused by a genetically determined anomaly of craniofacial development, rather than by local factors influencing alveolar growth. The article highlights the importance of cephalometric analyses in identifying other derangements within the cranial base and facial skeleton [44].

Other studies have investigated genetic factors that contribute to the development of open bite, and have found that genes involved in the growth and development of bones, teeth, and soft tissues may be associated with this malocclusion [45]. Several genes have been identified, including CYP19A1, GHR, TNF-α, RANKL/RANK, OPG, MYO1H, MMP, TIMPs, α-actin, and PTHR1 [46]. However, environmental factors are also important, and the occurrence of open bite is multifactorial, resulting from the interaction of genetic and environmental factors. Habits such as pacifier sucking and digital sucking are among the most prominent environmental variables involved in the establishment of open bite.

According to Nishio and Huynh [47], there is evidence to suggest that genetics play a role in the development of open bite. One study mentioned in the document found that two candidate genes, PAX5 and ABCA4-ARHGAP29, have been associated with vertical discrepancies ranging from skeletal deep to open bite. Additionally, another study found that patients with deep bite and the hypodivergent phenotype had a significant occurrence of palatally displaced maxillary canines, which suggests a genetic component to the etiology of this dental anomaly. However, further studies are necessary to clarify the frequency of occurrence of these dental disturbances in patients with vertical skeletal malocclusions and whether there is a genetically aetiological association between these disorders. A cross-sectional study aimed to evaluate whether polymorphisms of four genes (TNF-α, MTR, MTRR, and TGFβ1) could be biomarkers for oral health-related quality of life (OHRQoL) in preschoolers with anterior open bite (AOB). Overall, the study provides valuable insights into the genetic background of AOB and its impact on OHRQoL in preschool children [48].

In our research, we will employ a variety of advanced genetic analysis techniques to unravel the complex etiology of open bite. Genome-Wide Association Studies (GWAS) will allow for a comprehensive exploration of the entire genome, revealing specific genetic loci associated with open bite malocclusion and offering crucial insights into the genetic basis of this condition. Epigenome-Wide Association Studies (EWAS) will provide an in-depth analysis of epigenetic modifications, such as DNA methylation and histone modifications, which significantly influence open bite development by shaping gene expression patterns. Furthermore, through RNA sequencing (RNA-seq) analysis, we will seek to identify genes that exhibit differential expressions in tissues related to open bite, providing valuable insights into the molecular mechanisms underlying this condition and uncovering potential therapeutic targets. The integration of GWAS data with Expression Quantitative Trait Loci (eQTL) analysis will offer a comprehensive approach to understanding the intricate relationship between genetics and the manifestation of this dental condition. These genetic analysis methods will be pivotal in advancing our knowledge of open bite etiology and will hold promise for innovative prevention and treatment strategies.

## 10. Treatment

The degree and form of malocclusion, the dysfunction, the age of the patient, and the general well-being, along with other individualized variables, all influence open bite therapy. Orthodontic therapy is generally considered the first line of treatment for open bite, and may involve the use of braces, clear aligners, or other orthodontic devices to reposition the teeth correctly and close the open space. In some cases, a surgical approach may be essential to address the fundamental skeletal problem responsible for the open bite.

There are different treatment strategies depending on the type of dysgnathy or dentoalveolar malformation, age, and desired treatment goals in terms of function, aesthetics, and stability:

### 10.1. Myofunctional Therapy

**Figure 13:**
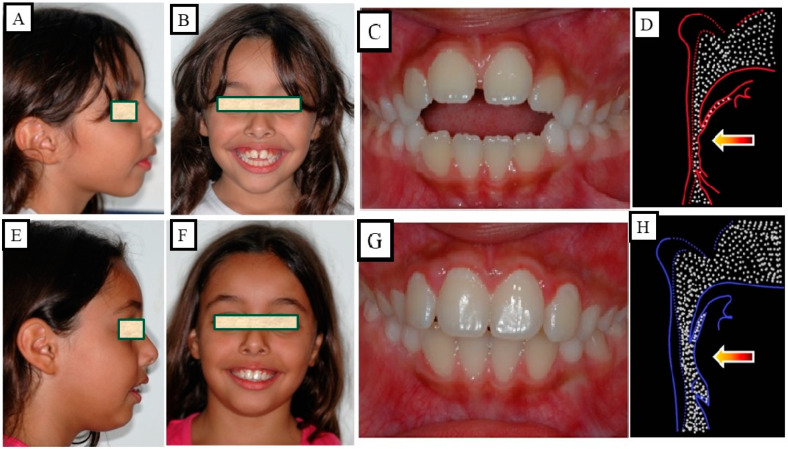
Patient with an anterior open bite. Due to the narrowed tongue space in the upper jaw and, thus, narrowed airways, there was a developmental disorder of the alveolar bone and the teeth. (**A**–**D**) Situation before treatment, (**D**) shows the narrowed airway. (**E**–**H**) The extra-oral and intraoral images after treatment, there was spontaneous bite closure after maxillary expansion. The physiological tongue position led to an expansion of the airways.

The aim of this treatment is to normalize function. The most common example is unphysiological breathing due to the position of the tongue in the oral cavity. This disorder leads to a narrowing of the airways, which causes an unphysiological tongue position. This malposition of the tongue can cause growth and tooth eruption disorders. With this treatment strategy, the function changes as well as the occlusion and the profile (Figure 13A–H).

### 10.2. Skeletal Treatment by Influencing Growth

**Figure 14:**
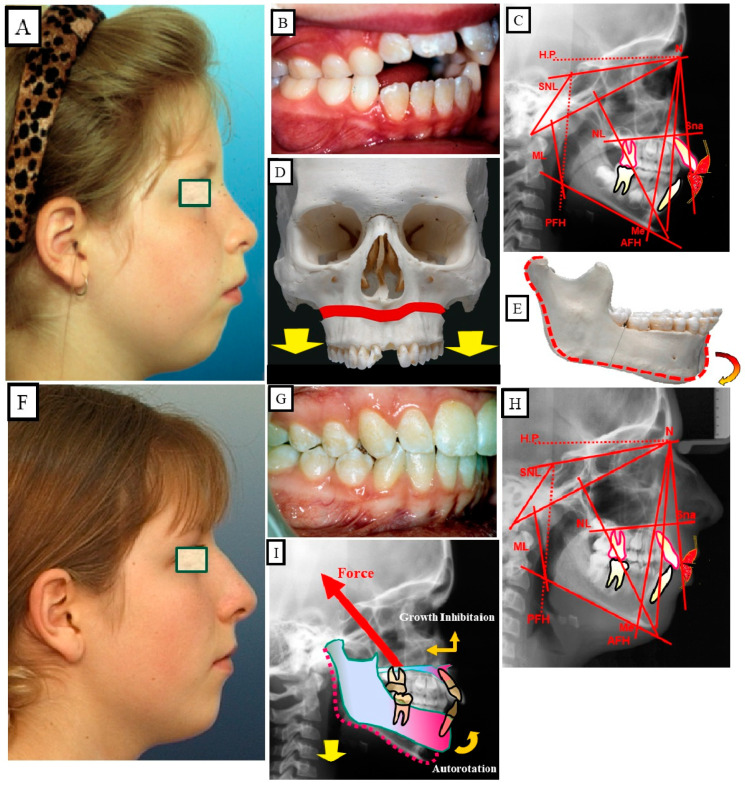
Skeletal open bite in a 10-year-old female patient, treated with growth-influencing measures. (**A**–**C**) Profile photo shows a long face with a skeletal open bite, which is confirmed intraorally and skeletal or cephalometrically. (**D**,**E**) Overdevelopment of the maxilla and alveolar bone resulted in posterior rotation of the mandible and thus posterior displacement of the mandible, resulting in the lower face lengthening. (**F**–**H**) treatment results of influencing the growth of the upper jaw in the vertical dimension, a harmonization of function and form, and, thus, a change in aesthetics. (**I**) Representation of the growth inhibition of the maxilla in the vertical direction, the autorotation of the mandible, and the alteration in position of the mandible.

The existence of sufficient growth is a prerequisite for this treatment strategy. The growth of the maxilla can be reduced by vertical forces so that the mandible autorotates when the mandibular growth is undisturbed. This autorotation changes the position of the mandible in the vertical and sagittal dimensions. This leads to a reduction in the lower facial height and closure of the open bite. With this treatment strategy, the function changes as well as the occlusion and the profile (Figure 14A–I).

### 10.3. Skeletal Treatment through Orthognathic Surgery

**Figure 15:**
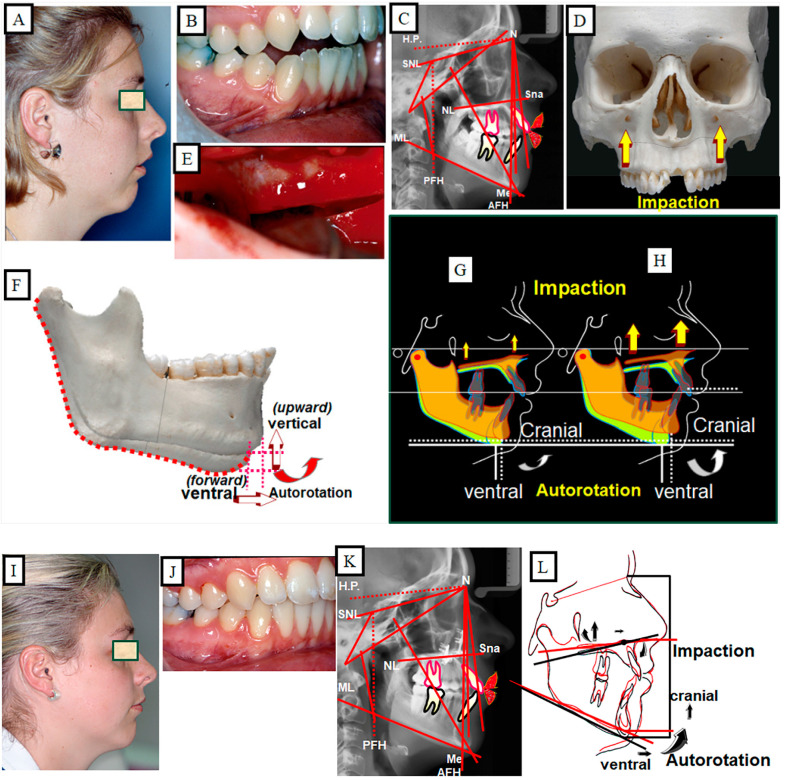
Skeletal open bite of a 25-year-old patient treated by orthognathic surgery. (**A**–**C**) The photograph shows a long face with a skeletal open bite, which is confirmed intraorally and is skeletal or cephalometric. (**D**,**E**) To correct the vertical relation, impaction of the maxilla was performed, and the excess bone of the maxilla was reduced. (**F**) A result of the maxillary action, the mandible autorotates with a change in sagittal and vertical position. (**G**,**H**) Simulation of the surgical impaction of the maxilla and the reaction of the mandible as described with cranial and simultaneous ventral autorotation. The greater the impact, the greater the autorotation of the mandible. (**I**–**K**) Situation after the treatment. (**L**) Superposition of the cephalograms pretreatment (black) and posttreatment (red).

A prerequisite for this treatment strategy is completed growth. The skeletal open bite is corrected by surgically changing the vertical emission (upper jaw impaction), thus shortening the height of the lower face. A precisely planned maxillary impaction results in vertical and ventral autorotation of the mandible (Figure 15A–L).

**Figure 16:**
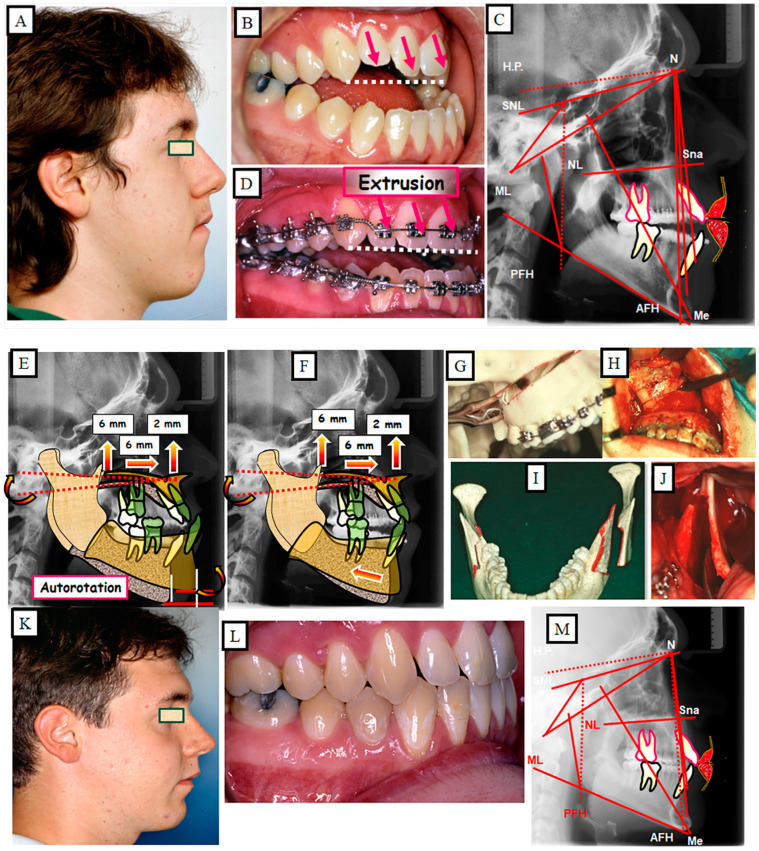
Skeletal and dental open bite in a 23-year-old man treated by orthognathic surgery. (**A**–**C**) Situation before the treatment, extended lower face due to the skeletal structure. The open bite has additionally strengthened the infra occlusion of the front teeth. (**D**) During the pre-surgical preparation, part of the open bite was corrected by extruding the front teeth. (**E**,**F**) Simulation of the surgical procedure. After impaction of the maxilla, the mandible autorotated with a change in position in the vertical and sagittal planes (**E**); for the final correction of the skeletal disgnathia, a dorsal displacement of the mandible (**F**) was carried out. (**G**,**H**) Description of maxillary impaction. (**I**,**J**) Representation of the surgical adjustment of the mandible for the correction of the position of the mandible. (**K**–**M**) Situation after the treatment.

Presurgical preparation sometimes requires dentoalveolar corrections by extrusion of the anterior teeth for a stable and perfect treatment result (Figure 16A–M). With this treatment strategy, the occlusion and the profile change, in addition to the function, are improved.

### 10.4. Dentoalveolar Compensation Therapy (Camouflage Therapy) of Skeletal Dysgnathia

**Figure 17:**
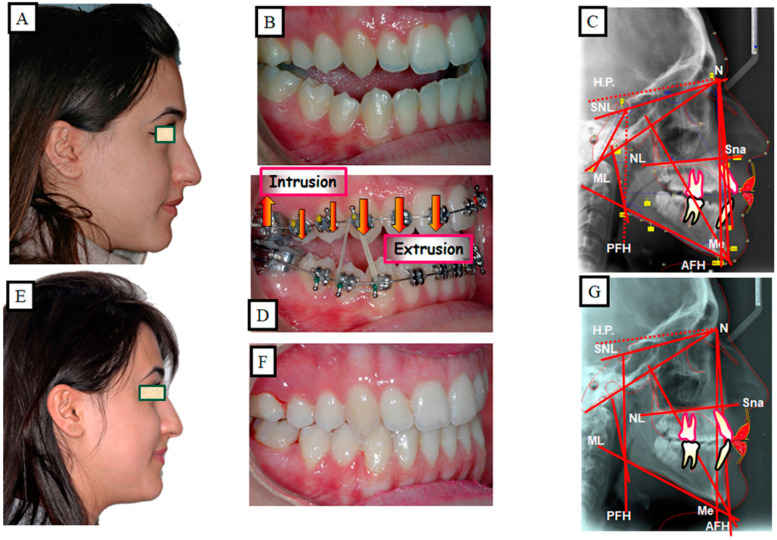
The Skeletal and dental open bite in a 25-year-old patient, treatment is dentoalveolar. It is crucial that the patient does not have any functional or aesthetic disorders extra-orally. (**A**–**C**) Situation before the treatment. (**D**) Presentation of the biomechanics used for ventoalveolar closure of the open bite; extrusion of the front and intrusion of the molars. (**E**–**G**) Situation after the treatment.

Some of the prerequisites for this therapy are that there are no functional disorders (such as difficult lip and mouth closure) and no serious extra-oral impairments (aesthetic disorders). This therapy uses intrusive biomechanics of the posterior teeth and extrusion biomechanics of the anterior teeth (Figure 17A–G). With this treatment strategy, the occlusion and the dental aesthetics change in addition to the function.

### 10.5. Dentoalveolar Treatment Strategy of the Dental Open Bite

**Figure 18:**
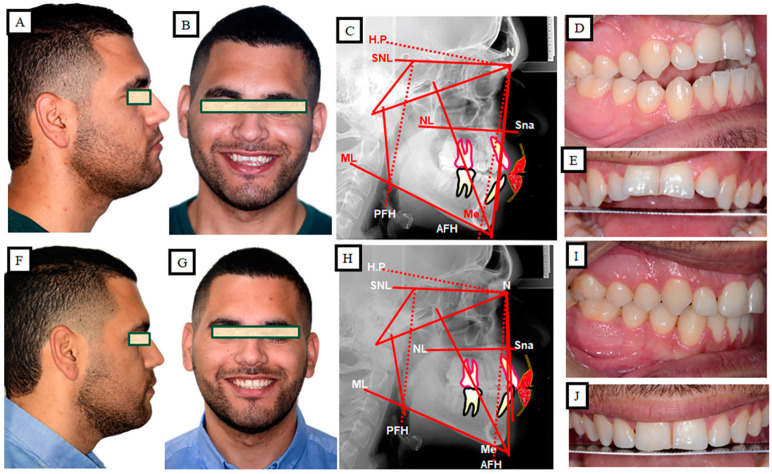
Dentoalveolar open bite in a 27-year-old patient, the treatment is dentoalveolar. The patient has no extra-oral functional or aesthetic disorders. There were intraoral dental functional and aesthetic disorders. (**A**–**E**) Before treatment, the infraocclusion of the front teeth in the upper jaw is clearly visible (**E**). (**F**–**J**) After treatment, to close the open bite, the maxillary front was extruded (**J**).

No abnormalities can be seen extra-orally because the harmony is in the bones and in the soft tissue structures. Therefore, the treatment focuses on the dental malformations. The most common dental malformation is infraocclusion of the front teeth. When smiling, these patients show an inverted smile compared to an ideal smile. With this treatment strategy, the occlusion and the dental aesthetics change in addition to the function (Figure 18A–J).

In addition to the aforementioned factors, significant advancements in technology have brought about promising prospects in the realm of malocclusion treatment. Innovations including technologies like 3D printing and computer-assisted design and manufacturing (CAD/CAM) have revolutionized the field by enabling the production of personalized orthodontic appliances and surgical guides. This breakthrough facilitates the attainment of enhanced treatment precision and efficiency, as the appliances can be tailored to meet the specific requirements of each patient. The utilization of these cutting-edge technologies holds great potential in optimizing treatment outcomes for individuals with malocclusion.

However, it is important to recognize that achieving the best possible results in malocclusion treatment necessitates the development of a personalized treatment plan under the guidance of a qualified orthodontic specialist. Several crucial factors must be taken into account, including the age and general well-being of the patient, as well as the degree and form of the malocclusion. By carefully considering these aspects, orthodontists can tailor treatment approaches to suit the unique needs and expectations of each patient. Therefore, fostering open and effective patient–orthodontist communication is essential, as it ensures the establishment of a comprehensive treatment plan that aligns with the individual’s specific circumstances, leading to optimal outcomes.

## 11. Exploring the Etiology of the Open Bite through A Collaborative Cross Mouse Model

Dental conditions like open bite have significant implications for dental and overall wellness. To develop effective treatment strategies, it is imperative to comprehend the fundamental genetic and environmental variables that contribute to these conditions. In recent years, the utilization of mouse models, including the Collaborative Cross population, has emerged as a powerful method for investigating complex features and deciphering the underlying causes of dental problems. This section delves into how such creative methodologies can provide insights into the phenotypes of open bite, shedding light on underlying causes as well as prospective treatment methods [49].

## 12. Unveiling the Genetic Basis

Animal models provide an excellent platform for studying the genetic variables that contribute to deep bite and open bite. Scientists can develop mice with oral characteristics similar to these disorders by altering certain genes or causing mutations. This enables the study of potential genes implicated in the formation and preservation of dental occlusion. Scientists can investigate the impacts of specific genes and their connections on dental morphology and occlusal relations using knockout or knock-in mice models. Such investigations provide valuable insights into the genetic basis of deep bite and open bite, expanding our understanding of the complex genetic networks underlying these conditions [50].

The Collaborative Cross (CC) mouse population offers an exceptional chance to explore the multidimensional character of the open bite. The CC population is made up of genetically varied recombinant inbred mouse strains descended from many founder strains. This genetic diversity allows scientists to examine the effects of variation in genetic backgrounds on phenotypic variability. By thoroughly phenotyping the dental occlusion of CC mice, researchers can identify genetic loci associated with deep bite or open bite traits. Subsequent mapping studies help pinpoint specific genomic regions and candidate genes contributing to the observed phenotypes, enabling a more comprehensive understanding of the underlying biological mechanisms [51,52,53,54,55,56].

## 13. Discussion

Open bite malocclusion is a complex dental condition that can have genetic, environmental, and developmental causes. Genetic factors have an important impact on the development of open bite, involving certain genes and signaling pathways that play a part in jaw development, the eruption of teeth, and dental occlusion. Epigenetic mechanisms like DNA methylation and microRNA regulation may additionally impact malocclusion progression. Recognizing the genetic and epigenetic elements involved in open bite can lead to the development of better prevention and treatment strategies.

The treatment of open bite depends on various factors, and orthodontic treatment is generally the first line of approach. This may involve the use of braces, clear aligners, or other orthodontic appliances to move the teeth into the correct position and close the open space. In some cases, surgical intervention may be necessary to address the underlying skeletal issue causing the open bite. Advancements in technology, such as 3D printing and CAD/CAM, have improved treatment precision and efficiency.

Research using animal models, particularly the Collaborative Cross mouse population, has provided valuable insights into the genetic components of malocclusion. The genetic diversity of the CC mouse population allows researchers to study the genetic variants that contribute to open bite and understand its underlying mechanisms. Identifying the genetic factors involved in open bite can lead to the development of more targeted and effective treatment approaches.

Beyond genetic factors, environmental influences play a pivotal role in the development of dental occlusion abnormalities. The Collaborative Cross mouse approach provides researchers with the means to study the interactions between genes and the environment by exposing CC mice to various environmental conditions. Scientists can test how external variables interact with genetic predispositions to alter dental occlusion by altering factors such as food, mechanical loading, or hormone effects. This holistic approach allows for a deeper exploration of the intricate interaction between genetic and environmental variables in the formation of open bite [49].

**Figure 19:**
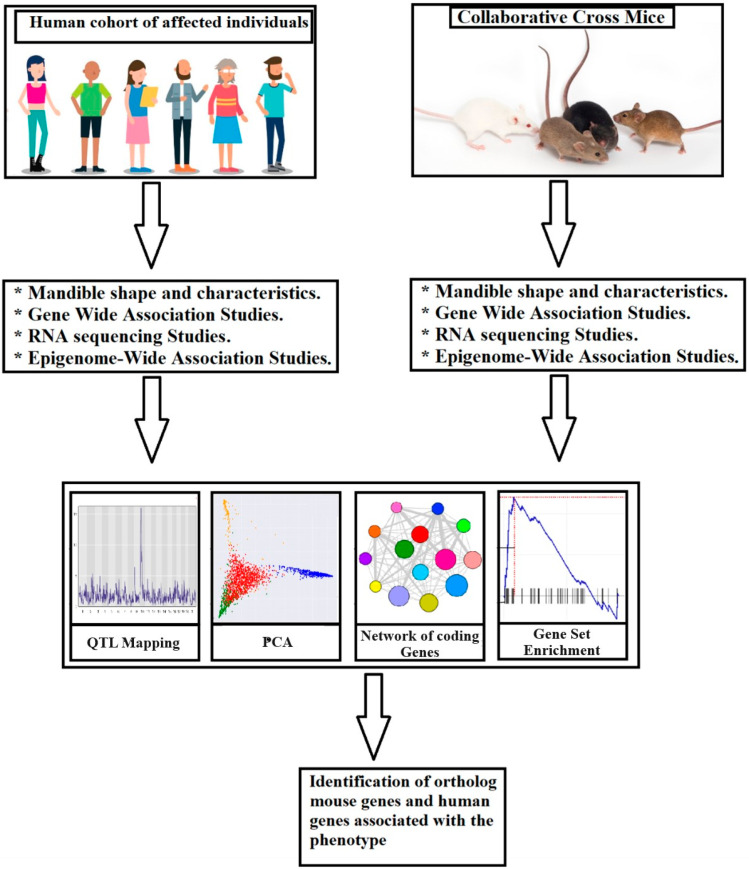
Process for creating system genetic datasets using cellular, molecular, and clinical trait data in order to investigate relationships between malocclusion and open bite phenotypes. Using QTL mapping in the CC mouse model and humans, regulatory genomic areas associated in phenotypic variance monitoring characteristics may be discovered by integrating SNP genotype data and RNA expression. The combination of existing data with future candidate gene association studies in people has the potential to find susceptibility genes linked to the development of open bite malocclusion in humans.

The findings from mice models and CC populations hold immense potential for improving diagnostic and therapeutic strategies in human dentistry. Understanding the genetic and environmental variables that contribute to deep bite and open bite in mouse models can enable scientists to find possible biomarkers and genetic susceptibility factors in humans. These results can help to guide the creation of focused therapies and tailored treatment plans. Moreover, mouse models facilitate the preclinical testing of novel therapeutic interventions, including gene therapies and pharmacological treatments, before advancing them to clinical trials. This translational approach ensures a more comprehensive and evidence-based approach to addressing the challenges posed by deep bite and open bite [49]. Figure 19 demonstrates the procedure for creating system genetic databases using cellular, molecular, and clinical trait data in order to investigate associations between malocclusion and open bite phenotypes [57]. The regulatory genomic regions linked in phenotypic variance monitoring characteristics may be discovered using QTL mapping in the CC mouse model and humans by merging SNP genotype data and RNA expression. Combining existing data with future potential gene association studies in people offers the potential to find vulnerability genes linked to the development of open bite malocclusion in humans.

## 14. Conclusions

Orthodontists find it challenging to correct open bite malocclusion. Functional appliances and adult surgery are two common treatment techniques for growing children. Fixed orthodontics and other habit-breaking appliances are effective treatments for minor cases. With this kind of malocclusion, relapse rates are higher. The stomatognathic system’s ability to function effectively is compromised in such circumstances. Since any mistake in determining the etiology could have negative consequences, more care should be given when diagnosing and arranging therapy for situations like this. As we delve into the intricate genetic underpinnings of open bite malocclusion, emerging tools and models like Collaborative Cross (CC) mice offer a promising avenue for research. The utilization of genetic insights and CC mice can lead to a deeper understanding of the genetic components governing this condition. By elucidating these genetic factors and their interactions, we pave the way for more precise diagnostic methods and targeted therapies. In this evolving landscape of malocclusion research, a paramount consideration is careful and meticulous diagnosis and treatment planning. Given the potential repercussions of errors in determining etiology, practitioners should exercise the utmost diligence. With genetics and CC mice at the forefront of exploration, the path toward innovative preventive and therapeutic strategies for open bite malocclusion is being illuminated, heralding a new era of dental care.

## Data Availability

This study did not report any data.
